# High-Sensitivity C-Reactive Protein and Cardiovascular Disease Across Countries and Ethnicities

**DOI:** 10.6061/clinics/2016(04)11

**Published:** 2016-04

**Authors:** Francisco Antonio Helfenstein Fonseca, Maria Cristina de Oliveira Izar

**Affiliations:** Universidade Federal de São Paulo, Departamento de Medicina, Divisão de Cardiologia, São Paulo/SP, Brazil

**Keywords:** C-reactive Protein, Ethnic Groups, Risk Factors, Cardiovascular Disease

## Abstract

Despite substantial differences in ethnicities, habits, cultures, the prevalence of traditional cardiovascular risk factors and affordable therapies, atherosclerosis remains the major cause of death in developing and developed countries.

However, irrespective of these differences, inflammation is currently recognized as the common pathway for the major complications of atherosclerosis, stroke, and ischemic heart disease. A PubMed search was conducted for “high-sensitivity C-reactive protein” (hs-CRP) in combination with the terms race, ethnicity, gender, prevalence, geographic, epidemiology, cardiovascular, obesity, diabetes, hypertension, cholesterol, smoking, ischemic heart disease, stroke, and mortality. This review includes the articles that pertained to the topic and additional articles identified from the reference lists of relevant publications.

This review describes the marked differences in cardiovascular mortality across countries and ethnicities, which may be attributed to inequalities in the prevalence of the classic risk factors and the stage of cardiovascular epidemiological transition. However, hs-CRP appears to contribute to the prognostic information regarding cardiovascular risk and mortality even after multiple adjustments. Considering the perception of cardiovascular disease as an inflammatory disease, the more widespread use of hs-CRP appears to represent a valid tool to identify people at risk, independent of their ancestry or geographic region. In conclusion, this review reports that the complications associated with vulnerable atherosclerotic plaques are triggered by the major mechanisms of dyslipidemia and inflammation; whereas both mechanisms are influenced by classic risk factors, hs-CRP contributes additional information regarding cardiovascular events and mortality.

## INTRODUCTION

Currently, the link between inflammation and cardiovascular disease (CVD) appears to be firmly established 1,2-3. Among the several biomarkers that have been proposed for cardiovascular risk stratification, high-sensitivity C-reactive protein (hs-CRP) appears to contribute to the identification of people at risk of developing CVD 4,5,6,7-8; however, the evaluation of hs-CRP has not yet been widely recommended in guidelines 9,10-11. Although some differences in the absolute event rates in patients with similar hs-CRP levels have been reported between genders and ethnicities 12,13, the reliability of hs-CRP determination appears to be valid for all patients, even after multiple adjustments 14. In light of the increased cardiovascular mortality in developing countries, a critical analysis of the available research on hs-CRP with respect to different countries and ethnic groups appears warranted.

### CRP Levels, Ethnicities, and Cardiovascular Events South and East Asians

In the Japan Collaborative Cohort study 15, subjects from 45 communities in Japan (13,282 men and 24,487 women) were followed for up to 13 years. After multiple adjustments, the hs-CRP levels in the highest quartile (>0.85 mg/L) versus the lowest quartile (<0.19 mg/L) were positively associated with CVD in men. In women, the hs-CRP levels in the highest quartile (>0.93 mg/L) versus the lowest quartile (<0.19 mg/L) were also positively associated with CVD (Table 1).

In the Hisayama study 16, a Japanese population-based, prospective cohort study, 2,589 participants were followed for 14 years. The annual incidence rates of coronary heart disease (CHD) progressively increased with increasing hs-CRP levels. The risk of CHD in the highest quartile group was 2.98-fold higher than the risk in the lowest quartile (Table 1).

Among the Chinese participants (n=1,847) enrolled in the Hong Kong Cardiovascular Risk Factors Prevalence Study 2 (CRISPS 2) 17 and followed for a median of 9.4 years, the subjects with incident CVD had higher median (interquartile range [IQR]) hs-CRP levels (1.28 [0.63–2.42]) than those without CVD (0.68 [0.33–1.47]); *p*<0.001 (Table 1).

A meta-analysis that included South and East Asian populations showed differences in the hs-CRP levels between these populations. Although the median hs-CRP level was 2.63 mg/L among the South Asian population, a lower median level (0.97 mg/L) was found among the East Asian population 18.

In the Indian Atherosclerosis Research study 19, hs-CRP levels were quantified in 774 subjects with coronary artery disease (CAD) and 1,544 asymptomatic individuals. During the 4-year follow-up period, the patients in the highest quartile of hs-CRP level (>3.58 mg/L) had a 4-fold higher risk for coronary events than patients in the first quartile (<0.7 mg/L) (Table 1).

Taken together, these studies suggest that the hs-CRP levels in these populations may vary according to the geographic region; however, despite these differences, the hs-CRP levels remained related to cardiovascular risk.

### Africans and African-Americans

An interesting study compared the hs-CRP levels between African-Americans (n=1,510) from the US and Africans from Nigeria (n=1,254) 20. In this cross-sectional analysis, the hs-CRP levels were higher among the African-Americans than among the Nigerians, suggesting differences among related ethnic groups living in countries at different stages of development.

The Profiles of Obese Women with the Insulin Resistance Syndrome (POWIRS) study included African (n=102) and Caucasian (n=115) women from South Africa 21. Higher mean hs-CRP levels were observed among the African women (4.91 mg/L) than among the Caucasian women (2.99 mg/L), suggesting differences in the hs-CRP levels between two different ethnic groups living in the same country.

Differences in hs-CRP levels were also observed in the Study of Women's Health Across the Nation (SWAN) study 22, which included 3,154 women in the United States without prior CVD. African-American women had the highest median hs-CRP levels (3.2 mg/L), followed by Hispanic (2.3 mg/L), white (1.5 mg/L), Chinese (0.7 mg/L), and Japanese (0.5 mg/L) women.

Similar results were found in the Multi-Ethnic Study of Atherosclerosis (MESA) study in the US; higher mean hs-CRP levels were detected among African-Americans (4.34 mg/L), followed by Hispanics (4.06 mg/L), Caucasians, (3.32 mg/L) and Chinese individuals (1.73 mg/L). According to the findings of the MESA study, hs-CRP levels were predictive of CVD only in Caucasians 23.

In a meta-analysis involving 18,585 subjects of African ancestry (African-Americans, African-Caribbeans, and Africans from Nigeria and Kenya), hs-CRP levels were higher in the African ancestry group (geometric mean, 2.60 mg/L) than among Hispanics (2.51 mg/L, n=5,049), South Asians (2.34 mg/L, n=1,053), Caucasians (2.03 mg/L, n=104,949), and East Asians (1.01 mg/L, n=39,521) 18.

In the Reasons for Geographic and Racial Differences in Stroke (REGARDS) study 24, African-Americans (n=7,853) had a median (IQR) hs-CRP of 2.8 (5.1) mg/L, which was higher than the level observed in the Caucasian population (n=11,238) with a median (IQR) hs-CRP of 1.8 (3.2) mg/L.

### Caucasians

The Whitehall II study 25, a long-term prospective cohort of 7,350 British civil servants, examined the relationship between baseline hs-CRP levels and outcomes during a median follow-up of >14 years. The baseline CRP levels (median [IQR]) were higher among participants who had fatal CVD events (1.49 [2.47] *vs* 0.84 [1.30] mg/L; *p*<0.0001) than among controls.

In the MESA study 23, Caucasian participants (n=2,362) were followed for a mean of 4.6 years. A positive association was observed between the hs-CRP levels and cardiovascular events (hazard ratio [HR], 1.23; 95% confidence interval [CI], 1.04–1.47; *p*<0.01).

In the Diabetes Heart Study 26, baseline hs-CRP levels were measured in 846 subjects with type 2 diabetes who were followed-up for a mean (standard deviation [SD]) of 7.3 (2.1) years. By the end of the study follow-up, the patients with baseline hs-CRP levels of 3–10 mg/L were 2 times more likely to be deceased and the patients with hs-CRP levels >10 mg/L were 5 times more likely to be deceased.

In the Physicians' Health Study 4 that evaluated apparently healthy and predominantly white male physicians in the US, hs-CRP was related to adverse cardiovascular outcomes. The subjects in the highest quartile of hs-CRP (≥2.11 mg/L) had a 3 times higher incidence of myocardial infarction and 2 times higher incidence of ischemic stroke than the subjects in the lowest quartile (≤0.55 mg/L). In the Women's Health study 5 that involved predominantly white women, the participants in the highest hs-CRP quartile (median, 8.5 mg/L) had 4.4 times more cardiovascular events than those in the lowest hs-CRP quartile (median, 0.6 mg/L; *p*<0.001).

### Hispanics

The Justification for the Use of Statins in Prevention: an Intervention Trial Evaluating Rosuvastatin (JUPITER) trial 27,28 was a primary prevention study that included 12,683 white and 5,117 non-white subjects with low-density lipoprotein (LDL) cholesterol levels <130 mg/dL (3.4 mmol/L) and hs-CRP levels ≥2 mg/L. The non-white population in the study included 2,224 blacks, 2,261 Hispanics, and 632 Asians or members of other ethnic groups. The non-white population had higher median (IQR) baseline hs-CRP levels than the white population (5.1 [3.2–8.8] *vs* 4.0 [2.7–6.5] mg/L). Additionally, the baseline median IQR hs-CRP levels were higher among blacks than in the other ethnic groups (blacks, 6.2 [3.7–11.1] mg/L; Hispanics, 4.4 [2.9–7.1] mg/L; and whites, 4.0 [2.7–6.5] mg/L). Whereas all 3 ethnic groups had the same age and body mass index (BMI) profiles, differences were demonstrated in their hs-CRP levels. The event rates for the whites and non-whites (Hispanics and blacks) per 100 person-years of follow-up were 1.44 and 1.11 in the placebo groups, respectively. After multiple adjustments, these results were considered similar for both of the ethnic groups, suggesting that the higher hs-CRP levels among the blacks and Hispanics may not be related to the same increase in the absolute risk observed in the Caucasians.

### Cardiovascular Risk Factors Related to Hs-CRP by Countries and Ethnicities Obesity

Obesity is related to hypertension, diabetes, CVD and to a chronic inflammatory state. In the Canadian Health Measures Survey 29, hs-CRP levels were evaluated in 1,805 subjects. The mean (standard error) hs-CRP levels were 0.65 (1.15), 1.26 (1.16), and 2.28 (1.16) mg/L in normal-weight, overweight, and obese subjects, respectively.

A systematic review and meta-analysis 30 that included 51 cross-sectional studies (76,444 American/European and 59,040 Asian subjects) confirmed the relationship between hs-CRP and BMI as well as the relationship with waist circumference and waist-to-hip ratio. This correlation was higher among women (0.53 [95% CI, 0.45–0.60]) than men (0.24 [95% CI, 0.19–0.29]) in the North American/European populations. The correlation observed in these ethnic groups was consistently higher than that observed in the Asian population.

Marked differences in the prevalence of obesity were observed across countries from Europe, Asia, Africa, and the US. Furthermore, according to the World Health Organization (WHO) 31, the lowest prevalence of obesity was reported in some countries in Eastern and Southern Asia and countries from Eastern or Central Africa. Conversely, a high prevalence of obesity has been reported in few countries in Africa, whereas it was widely reported in European countries and the US.

### Diabetes

In a cross-sectional study involving 1,730 Chinese subjects, a glucose tolerance test was performed and the participants were classified as having normal glucose tolerance (NGT, n=1,258), impaired fasting glucose (IFG, n=126), or impaired glucose tolerance (IGT, n=346) 32. After multiple adjustments, the odds ratios (ORs) across the quartiles of hs-CRP remained associated with IFG and IGT. The geometric mean ([SD]) levels for hs-CRP were 1.18 (2.48), 2.47 (3.52), and 2.42 (3.17) mg/L for NGT, IFG, and IGT, respectively.

In a cross-sectional study that included 822 men and 1,097 women in Japan, an association was observed between fasting glucose levels and hs-CRP, but only for women. The hs-CRP levels progressively increased with higher fasting glucose levels between 90 mg/dL (5.0 mmol/L) and 125 mg/dL (6.9 mmol/L), or known diabetes 33. An association between increasing hs-CRP levels and worsening insulin resistance was also reported in a South Asia (India) study 34.

In the US, the MESA study (n=6,067 subjects) 23 assessed the relationship between baseline hs-CRP and the incidence of diabetes during a mean follow-up of 4.6 years in 4 ethnic groups: Caucasians (38.9%), African-Americans (26.4%), Hispanics (22.3%), and Chinese-Americans (12.3%). A correlation was observed between hs-CRP and the incidence of diabetes (highest quartile vs first quartile; HR, 1.7; 95% CI, 1.3–2.4), and the association was similar across all the ethnic groups.

In the British population of the Whitehall II study, baseline hs-CRP levels were predictive of diabetes. After a mean follow-up of >14 years, the mean (SD) hs-CRP levels among subjects who developed diabetes were 1.44 (2.39) and 0.78 (1.21) mg/L in subjects who developed diabetes and those who remained free of diabetes, respectively 25.

High glucose levels have been reported in few countries in Asia (India and Pakistan) and Africa (Libya, South Africa, Nigeria, and Ghana), but were common in Eastern European countries, North America, Central America, and South America. The lowest prevalence of hyperglycemia has been reported in countries in Eastern Asia, Eastern and Central Africa, parts of Europe (Western, Northern, and Southern European countries), and in some countries in Central and South America 31.

### Hypertension

Increased hs-CRP levels are predictive of incident hypertension 35,36,37,38-39. A cross-sectional study in South Africa evaluated 836 black subjects and tested the relationship between hs-CRP levels and central blood pressure. Hs-CRP levels were correlated with central blood pressure; however, the associations did not persist after adjustments were made for age, mean arterial blood pressure, and BMI 40. These findings contrast with the results of studies in other ethnic groups 41,42. The relatively high incidence of chronic infections among the black African population may have contributed to the high hs-CRP levels 42. Conversely, another study involving obese white and black women in South Africa showed high mean (IQR) hs-CRP levels in patients with hypertension (3.50 [2.05–4.95] and 5.50 [3.04–7.95] mg/L, respectively), and both risk factors were related to arterial compliance 43.

The lowest prevalence of hypertension has been reported in East and South Asian countries, some regions of Europe (Western, Southern, and Northern European countries), and some countries in North and South America. The highest prevalence of elevated blood pressure has been reported in countries in Africa (all regions) and Eastern Europe and in some Central and South American countries 31.

### Smoking

In the Ludwigshafen Risk and Cardiovascular Health (LURIC) study (n=3,316), baseline hs-CRP levels were significantly higher in active smokers than in individuals who had never smoked (4.9 *vs* 2.7 ng/mL) 44. A cross-sectional survey conducted in 1,172 healthy men in the US revealed that CRP levels increased in a stepwise manner for patients who had never smoked, those who were former smokers, and those currently smoking, with geometric mean values of 1.0±2.5, 1.3±2.5, and 2.0±2.7 mg/L, respectively (*p*<0.001). Additionally, a positive association was observed between CRP levels and the number of cigarettes smoked per day (*p*<0.01) among current smokers 45. Similar results were reported among women in the US (n=340) in a nested case-control study. Baseline hs-CRP levels were significantly higher in women who were current smokers than in non-smoking women (median [IQR], 0.38 [0.18–0.83] *vs* 0.3 [0.13–0.57] mg/L, *p*<0.032), even after multivariate analysis (*p*= 0.034). Significant increases were observed in hs-CRP levels across women who had never smoked, former smokers, and current smokers (*p*=0.041) 46.

In a hypertensive population from Japan, after multiple adjustments CVD events were significantly higher among current smokers than among former smokers. Moreover, the CVD risk was higher in currently smoking women (HR, 6.1; 95% CI, 2.8–13.4; *p*<0.001) than in men (HR, 1.4; 95% CI, 0.7–2.8; *p*=0.41). The risk of CVD among current smokers was significantly higher in the highest quartile of hs-CRP levels than in the lower hs-CRP quartiles 47.

The lowest prevalence of smoking has been reported in Africa (all regions), some countries in Northern Europe, and North and Central America. The highest prevalence of smoking has been reported in some countries of Eastern and Southern Asia, Eastern, Western, and Southern Europe, and North America 31.

### Hypercholesterolemia

Despite the weak association between cholesterol levels and hs-CRP 48,49, hs-CRP determination is particularly important for risk stratification in the presence of relatively low cholesterol levels 6,27. Clearly, the association between cholesterol levels and coronary mortality was established based on observational studies 50,51. Additionally, however, several meta-analyses 52,53,54-55 have demonstrated the benefits of lowering LDL cholesterol to decrease total and cardiovascular mortality rates, as well as several other cardiovascular events. Thus, data from the WHO on hypercholesterolemia around the world are also provided based on the impact of this risk factor on cardiovascular mortality rates beyond that of other risk factors that may affect hs-CRP levels. Cholesterol levels >200 mg/dL (5.2 mmol/L) are common in several countries in all the European regions (Eastern, Western, Northern, and Southern Europe) and are also highly prevalent in the countries of North America. Conversely, a lower prevalence of hypercholesterolemia has been observed in Eastern and Southern Asia, all African regions, and in the majority of countries of Central and South America (with a few exceptions) 31.

### Mortality Rates Due to Ischemic Heart Disease and Stroke by Geographic Region

Marked differences in mortality rates due to ischemic heart disease have been reported among Asian countries with higher rates observed in Southern Asia 31.

**Figure 1:**
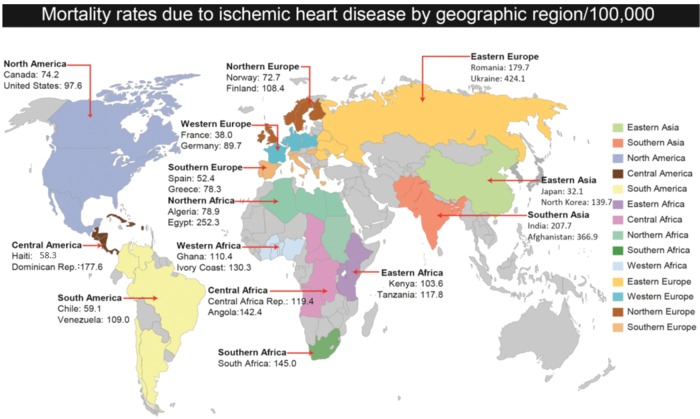
Estimated age-standardized death rates (per 100,000) due to ischemic heart disease in individuals of both sexes among Asian, American, African, and European populations according to the World Health Organization 31. Values for countries with the lowest and highest rates are shown per region.

Higher ischemic heart disease mortality rates have been observed in Eastern Europe. High mortality rates for this disease are also common in Africa, particularly in countries in the northern region. Conversely, lower mortality rates due to ischemic heart disease have been reported in countries in Western, Northern, and Southern Europe. These rates were not homogeneous in the Americas (Figure 1).

**Figure 2:**
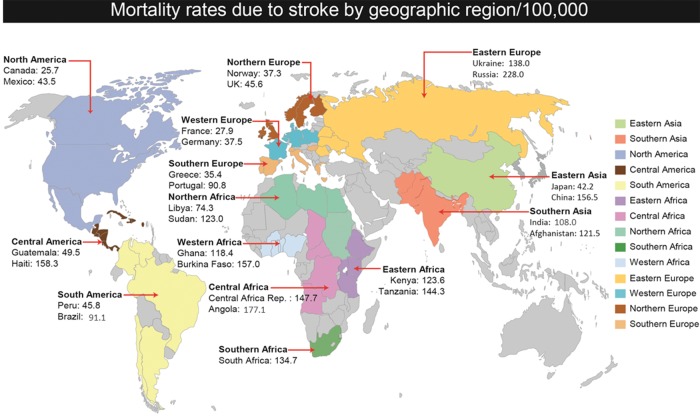
Estimated age-standardized death rates (per 100,000) due to cerebrovascular disease in individuals of both sexes among Asian, American, African, and European populations according to the World Health Organization 31. Values for countries with the lowest and highest rates are shown per region.

The highest mortality rates due to stroke have been observed among African countries (mainly in the eastern, central, and southern regions) and in Eastern Europe. High rates for stroke mortality have also been observed in some countries in Central and South America. In contrast, the lowest rates for stroke mortality have been observed among countries in the Western, Northern, and Southern European regions (except Portugal) (Figure 2).

### Significance of Hs-CRP for Risk Stratification

Increased global mortality is associated with atherosclerosis, considered an inflammatory disease 1,2-3,56, which appears to be independent of race, culture, or the country's development stage. According to the WHO, the highest ischemic heart disease mortality rates have been observed in Southern Asia, Eastern Europe, and Africa. The prevalence of cardiovascular risk factors across continents is heterogeneous; however, some regions with the highest hs-CRP levels also have the highest prevalence of hypertension, diabetes, and obesity. This association holds in Eastern Europe, a region that also shares a high prevalence of hypercholesterolemia. The high mortality rates in Africa due to ischemic heart disease appear to be related to the high prevalence of hypertension, hyperglycemia, and hypercholesterolemia. In Southern Asia, the high mortality rates due to ischemic heart disease appear to be related to the high prevalence of diabetes and hypercholesterolemia.

With regard to mortality rates due to stroke, the highest rates have been observed in Southern Asia, Africa, Eastern Europe, and a few countries in Central America, which are all regions with a high prevalence of hypertension. Interestingly, a recent analysis of the impact of low socioeconomic position (SEP) across the life span revealed that this condition is related to high hs-CRP levels in adulthood and appears to be at least in part independent of other metabolic or traditional risk factors 57,58.

Levels of hs-CRP appear to correlate with cardiovascular mortality rates, despite the marked differences in ethnicities and the stage of disease control.

Based on their hs-CRP levels, at-risk individuals have been identified in primary 59,60 and secondary 48,61 cardiovascular prevention trials. A large ongoing trial, the Canakinumab Anti-inflammatory Thrombosis Outcomes Study (CANTOS) 62, includes individuals with persistently high hs-CRP levels after myocardial infarction; it will evaluate whether the use of anti-inflammatory therapy reduces cardiovascular events.

### Variability of Hs-CRP Concentrations by Populations

In the large JUPITER trial, hs-CRP concentrations were determined in populations from Europe, Asia, Africa and the Americas at screening, before randomization, and after years 1, 2, 3, and 4 of the study. The median hs-CRP concentrations in the untreated population showed little change over these years, declining from 3.8 mg/L at randomization to 3.4 mg/L at 4 years. Thus, these results demonstrated a strong persistence of hs-CRP concentrations, even in a selected population of subjects with high baseline values. In the same study 63, these findings regarding repeated hs-CRP measurements were comparable to those observed for blood pressure and lipid variables.

Recently, the evaluation of six-year changes in hs-CRP concentrations was examined in the large Atherosclerosis Risk in Communities (ARIC) study 64. Compared with subjects with sustained low/moderate hs-CRP, those with increased or sustained high hs-CRP had increased risks of diabetes, coronary heart disease, ischemic stroke, heart failure and mortality.

The intra-individual variability of C-reactive protein was also tested in the Multi-Ethnic Study of Atherosclerosis (MESA) 65. Even after multivariable adjustment, the intraclass correlation coefficient (ICC) of hs-CRP was 0.62 (95% CI, 0.55-0.68), a value that was higher than the ICC observed for total cholesterol and non-HDL-cholesterol. However, due to its variability, the repeated measurement of hs-CRP appears more appropriate than the use of a single sample value.

An interesting study conducted in a Chinese population with type 2 diabetes revealed that hs-CRP concentrations correlated with arterial stiffness as a continuous variable, in multivariate models, even among individuals with low-grade inflammation 66. The prevalence of some classic risk factors, such as hypercholesterolemia and obesity, is lower in Japanese and Chinese populations, which may partially explain their lower levels of hs-CRP than those of other Asian populations. However, even in these populations with lower cardiovascular mortality rates, hs-CRP appears to be a continuous” variable related to cardiovascular risk.

Finally, another interesting consideration is the stability of CRP in stored samples. A recent study that examined hs-CRP levels over 11 years of storage at -80°C found a coefficient of correlation of 0.98, suggesting that under appropriate storage conditions, the measurement of hs-CRP is not affected over time 67.

**Figure 3:**
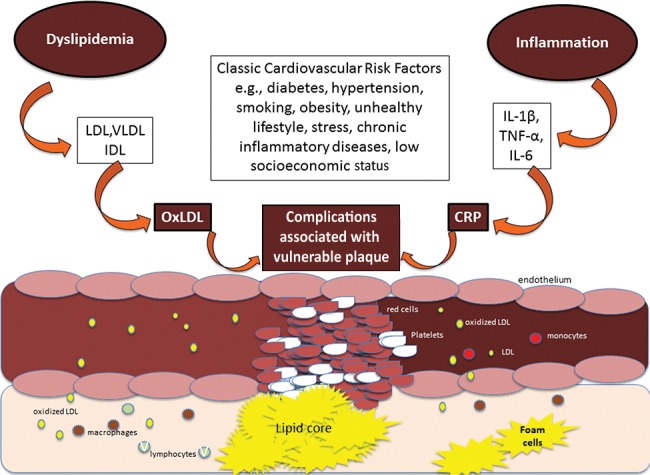
Increased cholesterol concentrations with oxidized lipoproteins and inflammatory stimuli through the release of cytokines with subsequent increase in C-reactive protein. Both pathways are related to classic risk factors and contribute to the development of and complications associated with vulnerable atherosclerotic plaques. Thus, lipid and high-sensitivity C-reactive protein determinants provide additional information regarding cardiovascular risk. Strategies to control both mechanisms appear germane to decreasing the global cardiovascular disease burden, independent of ethnicity or geographic region.

Marked differences in the cardiovascular mortality rates across countries and ethnicities may be attributed to inequalities in the prevalence of classic risk factors and the stage of the country's development. However, according to a robust meta-analysis, hs-CRP levels appear to provide additional prognostic information on cardiovascular risk and mortality, even after multiple adjustments 14. In fact, taking into account the perception of CVD as an inflammatory disease, the more widespread use of hs-CRP may represent an important tool to identify people at risk, independent of their ancestry or geographic region. The large ongoing CANTOS trial 62 involves subjects from different geographic regions and could answer important questions regarding the impact of anti-inflammatory therapy on the development of major adverse cardiovascular events (MACE). This study should also address whether a need exists for the establishment of different hs-CRP cut-off levels and the applicability of this biomarker for different ethnicities. Figure 3 summarizes the significance of the classic risk factors that influence the two major pathways related to cardiovascular disease, lipids and inflammation.

In conclusion, this review reports that complications involving the vulnerable atherosclerotic plaque are triggered by two major mechanisms, dyslipidemia and inflammation; although both are influenced by classic risk factors, each mechanism provides additional information regarding cardiovascular events and mortality.

## DECLARATION OF FINANCIAL SUPPORT/CONFLICTS OF INTEREST

The authors declare that no financial support was received for this work. Dr. Fonseca is a member of the CANTOS Global Steering Committee.

## AUTHOR CONTRIBUTIONS

Fonseca FA and Izar MC conceived the manuscript, reviewed the literature, and wrote the manuscript.

## Figures and Tables

**Table 1:** High-sensitivity C-reactive protein levels and cardiovascular disease events according to countries and ethnic groups.

Author	Country	Clinical trial	hs-CRP cut-off
Iso et al. (15) Hs-CRP levels were measured using an ultra-sensitive latex-enhanced immunoassay with an automatic analyzer (BN ProSpec Nephelometer; Dade Behring, Tokyo, Japan)	Japan	Japan Collaborative Cohort Study	*Lowest (<0.19 mg/L) vs highest (>0.85 mg/L) quartile* Correlated with cardiovascular mortality in men *Lowest (<0.19 mg/L) vs highest (>0.93 mg/L) quartile* Correlated with cardiovascular mortality in women
Arima et al. (16) Serum hs-CRP levels were analyzed using a modification of the Behring latex-enhanced CRP assay on a BN-100 nephelometer (Dade Behring) with a 2% interassay coefficient of variation	Japan	Hisayama study	*Lowest (<0.21 mg/L) vs highest (>1.02 mg/L) quartile* Correlated with coronary heart disease
Chow et al. (17) CRP was measured using a high-sensitivity, particle-enhanced immunoturbidimetric assay	China	CRISPS 2 study	*Comparison, >1 vs <1 mg/L* Correlated with cardiovascular disease
Rao et al. (19) Plasma hs-CRP levels were measured using the Roche Tina-Quant CRP (Latex) kit (Roche Diagnostics, Basel, Switzerland)	India		*Lowest (<0.7 mg/L) vs highest (>3.58 mg/L) quartile* Correlated with coronary events
Veeranna et al. (23) CRP was measured using the BN II Nephelometer (N High-Sensitivity CRP; Dade Behring)	United States	MESA study	hs-CRP levels correlated with cardiovascular disease only in Caucasians
Tabak et al. (25) CRP was measured using a high-sensitivity immunonephelometric assay with a BN ProSpec Nephelometer (Dade Behring, Eschborn, Germany)	United Kingdom		hs-CRP levels correlated with diabetes/fatal cardiovascular disease
Cox et al. (26)	United States		hs-CRP levels correlated with mortality in European Americans with type 2 diabetes
Ridker et al. (4) CRP was measured using an enzyme-linked immunosorbent assay (ELISA) based on purified protein and polyclonal anti–C-reactive protein antibodies (Calbiochem, San Diego, CA, USA)	United States		*Lowest (≤0.55 mg/L) vs highest (≥2.11 mg/L) quartile* Correlated with myocardial infarction and stroke in male physicians
Ridker et al. (27)	United States		*Lowest (median, 1.2 mg/L) vs highest (median, 4.4 mg/L) quartile* Correlated with cardiovascular events in women
Albert et al. (28)	Global		hs-CRP levels correlated with cardiovascular events in male and female participants in the JUPITER trial (independent of ethnic group)

All studies were observational (except the JUPITER trial).

hs-CRP: high-sensitivity C-reactive protein; CRISPS 2: Cardiovascular Risk Factors Prevalence Study 2; MESA, Multi-Ethnic Study of Atherosclerosis.
